# Impact of body mass index on semen parameters and reproductive hormones among men undergoing microsurgical subinguinal varicocelectomy

**DOI:** 10.1080/2090598X.2023.2206336

**Published:** 2023-05-09

**Authors:** Mohammed Mahdi, Ahmad Majzoub, Haitham Elbardisi, Mohamed Arafa, Kareim Khalafalla, Sami Al Said, Walid El Ansari

**Affiliations:** a Department of Urology, Hamad Medical Corporation, Doha, Qatar; b Department of Urology, Weill Cornell Medicine-Qatar, Doha, Qatar; c Department of Urology, Qatar University, Doha, Qatar; d Department of Andrology, Cairo University, Cairo, Egypt; e Urology Department, University of Texas McGovern Medical School Houston, Texas, USA; f Urology Department, MD Anderson Cancer Center, Houston, TX, USA; g Department of Surgery, Hamad Medical Corporation, Doha, Qatar; h College of Medicine, Qatar University, Doha, Qatar; i Department of Population Health, Weill Cornell Medicine-Qatar, Doha, Qatar

**Keywords:** Varicocele, micro-surgical varicocelectomy, male infertility, body mass index, obesity

## Abstract

**Background:**

Few studies assessed the relationships between BMI and post varicocelectomy semen quality and fertility potential and they reported inconsistent findings.

**Objective:**

To assess the association of BMI with semen parameters and reproductive hormones before and after microsurgical varicocelectomy.

**Materials and Methods:**

Retrospective chart review in a tertiary infertility center. Of 1170 patients with clinical varicocele during the study period (8 years), 813 patients were eligible and included. Patients were grouped into: Group A (kg/m^2^, *n* = 251 patients), B (BMI 25–29.9 kg/m^2^, *n* = 289), C (BMI 30–34.9 kg/m^2^, *n* = 183) and D (kg/m^2^, *n* = 90). Clinical data, semen parameters, sperm DNA fragmentation and hormonal profile were collected before and 3 months after microsurgical varicocelectomy.

**Results:**

Patients’ mean age was 35.87 ± 8.17 years. Higher-grade varicocele was significantly more prevalent in the lower BMI groups. BMI was significantly negatively correlated with preoperative sperm concentration, total motility progressive motility and total motile sperm count. Pre-operatively, sperm concentration, total motility, progressive motility and total motile sperm count showed significant differences between BMI groups, where higher BMI (Groups C and D) exhibited the poorest semen parameters. Postoperatively, all groups showed significant improvement in sperm concentration compared with pre-operative values. However, total and progressive motility were significantly improved in Groups A, B and C, while in Group D (highest BMI), total motility improved clinically but not statistically, progressive motility did not display improvement, and total motile sperm count was significantly improved only in Groups B and C. Postoperatively, mean improvements in semen parameters across the BMI groups were not significantly different, except for morphology, which improved significantly more in the less obese patients.

**Conclusion:**

For infertile patients with clinical varicocele undergoing micro-surgical varicocelectomy, BMI appears not to impact the improvements across most of the semen parameters and hormones. The procedure might improve the fertility potential.

## Introduction

Varicocele is defined as an abnormal dilatation of the pampiniform venous plexus of the spermatic cord. It affects around 15% of the general male population and 35%–80% of males with primary and secondary infertility respectively [1]. In patients with varicocele, abnormal seminal oxidative stress is considered one of the main pathophysiological mechanisms behind varicocele-induced infertility [2].

In addition, obesity is also a recognized risk factor for reduced male fertility [3–5]. It is associated with abnormal semen parameters brought about through several mechanisms similar to those implicated in varicocele-induced infertility including chronic inflammation, oxidative stress and sperm DNA fragmentation (SDF) [6].

Varicocele is considered a reversible and correctable cause of male factor infertility [7]. Current evidence strongly supports the improvement in semen parameters and fertility potential after varicocele repair [8–10]. Although many studies have examined the pre-operative clinical (including body mass index (BMI)) and laboratory factors predicting post-operative improvement in semen parameters and pregnancy outcome, there is no clear consensus about these factors [11–14].

To date, only seven studies have evaluated the association between obesity/BMI and post-varicocelectomy improvement of semen parameters. These studies reported inconsistent findings. Some found that BMI was an insignificant predictor of improvement in semen parameters after varicocelectomy [13–15]; whereas others confirmed a favorable outcome of the procedure on semen quality among patients with lower BMI [11,12,16,17]. Furthermore, most of these studies examined the association of BMI with postoperative semen parameters but not reproductive hormones [11,12,15,16], thus providing a restricted view of any possible relationship of the effect of BMI on hormonal profile which may affect spermatogenesis and alter the effect of varicocele repair. Only two studies assessed the association of BMI on the one hand and pre- and post-operative semen parameters and reproductive hormones on the other [13,17]. Such lack of knowledge is despite that among obese males, in addition to the altered sperm parameters, there is evidence of increased estradiol, and hypogonadism [18]. Research also suggests that obesity may alter the synchronized working of the reproductive-endocrine milieu, mainly the hypothalamic-pituitary-gonadal (HPG) axis along with its cross talks with other reproductive hormones [19]. Moreover, most studies employed modest sample sizes, varying from 35 to 143 patients [14,15]. Hence, the current evidence falls short of consensus, with inquires of limited scope and modest sample sizes. Surprisingly, to date, there is no systematic review or metanalysis on this important topic. These gaps in knowledge acted as the of the present research.

Therefore, the aim of the current study was to assess the association of BMI with semen parameters and reproductive hormones before and after microsurgical varicocelectomy among a large sample of infertile men. The specific objectives were to evaluate, for the same infertile men, the relationship between BMI on the one hand, and semen parameters (total motility, progressive motility, morphology, sperm DNA fragmentation) and reproductive hormones (estradiol, luteinizing hormone (LH), follicle stimulating hormones (FSH), prolactin, testosterone) on the other hand at two successive points in time, namely before and 3 months after the microsurgical varicocelectomy.

## Methods

### Study design, ethics and population

This retrospective study comprised a chart review of patients who presented with male factor infertility to our tertiary infertility center over a period of 8 years (1 January 2011 - 1 January 2019). The study was approved by the Medical Research Center (IRB) at our institution (MRC-1252/11), and a waiver of informed consent was provided. Infertile patients from different age groups with clinical varicocele who underwent microsurgical varicocelectomy were included. The exclusion criteria were history of genitourinary infection, exposure to heat, chemotherapy or radiotherapy, hormonal disturbances, the presence of genetic abnormality or prior infertility related treatments. Of 1170 total patients screened, 813 met the selection criteria and were included in the current analysis.

### Data collection

Data collected from patients’ electronic records included demographics, BMI and clinical data, results of semen analysis, SDF and reproductive hormonal profile [estradiol, follicle stimulating hormone (FSH), luteinizing hormone (LH), prolactin and testosterone. The data was retrieved from patient records at two time points: (1) at the initial infertility evaluation; and (2) again at the 3-month follow-up visit after the micro surgical varicocelectomy. Patients were grouped according to WHO international BMI categories into 4 groups: Group A (kg/m^2^, *n* = 251 patients), B (BMI 25–29.9 kg/m^2^, *n* = 289 patients), C (BMI 30–34.9 kg/m^2^, *n* = 183 patients) and D (kg/m^2^, *n* = 90 patients) [20].

### Study procedures

Semen tests were conducted on samples obtained via masturbation after 2–7 days of abstinence and analyzed according to WHO criteria (5th edition) [21]. SDF was measured using the sperm chromatin dispersion (SCD) method or ‘Halosperm kit’ (Halotech DNA, SL, Madrid, Spain) [22]. Static oxidation reduction potential (sORP) was assessed in unprocessed post-liquefied semen using the MiOXSYS (Caerus Biotechnologies, Geneva, Switzerland) [23]. The reported result is normalized by the sperm count and expressed as mV/10^6^ sperm/mL [24]. Analyses of reproductive hormones were performed at the same certified laboratory using third generation chemiluminescence immune assay on blood samples collected between 7 am and 10 am. The reference levels used were: FSH (1–19 IU/L), LH (1–9 IU/L), prolactin (73–407 mIU/L), total testosterone (10.4–35 nmol/L), and estradiol (73–275 pmol/L).

### Microsurgical procedure

Varicocelectomy was performed using the microsurgical subinguinal approach. All cases were done by the same urology team using a standardized approach [25]. Procedures were done under general anesthesia, utilizing a 2–3 cm subinguinal incision. After delivery of the spermatic cord, any dilated external spermatic veins were ligated. Following dissection of the external spermatic fascia, a surgical microscope (Pentero 900, Carl Zeiss Meditec, Jena, Germany) was used under × 18 magnification to explore the cord and dissect, separate, ligate (using titanium clips) and divide the internal spermatic veins. A micro Doppler probe was also used during the procedure to identify and preserve the testicular artery. Vas deferens and lymphatic vessels were preserved.

### Statistical analysis

Statistical analyses were performed using IBM Statistical Package for the Social Sciences (SPSS, version 25, Armonk, New York, U.S.). The normal distribution of variables was tested using histogram and Shapiro-Wilk test. Continuous variables were expressed by their mean ± standard deviation or median and interquartile range (IQR). Categorical variables were expressed by their frequencies and percentage. Spearman’s correlation was performed to explore the relationship between BMI with semen and hormone parameters. Wilcoxon signed ranks test was used to compare the results of the investigated parameters at two-time intervals (initially and 3 months following surgery). Kruskal–Wallis test was used to assess differences in preoperative semen and hormone results and compare the changes observed following varicocelectomy between the BMI groups. *P* values of 0.05 and less were considered statistically significant with an acceptable margin of error of 5%.

## Results

### Pre-operative characteristics of the sample

Mean age of the whole sample was 35.87 ± 8.17 years and mean BMI was 28.1 ± 5.8 kg/m^2^ (Table 1). Of the 813 patients included in the analysis, 673 had left clinical varicocele, and 140 had bilateral clinical varicocele. The Table also depicts the whole sample’s baseline levels of the semen parameters and reproductive hormones. Preoperatively, high-grade (grades II and III) left and bilateral varicoceles were significantly more prevalent among the lower BMI groups (Groups A and B, *p* < 0.001, data not presented).

**Table 1.** Preoperative characteristics of the sample(*N* = 813).

Parameter	Value
Demography (M ± SD)	
Age, years	35.87 ± 8.17
BMI, kg/m^2^	28.09 ± 5.83
Varicocele	
Side, n (%)	
Left	673 (83)
Bilateral	140 (17)
Max. vein diameter^*a*^, mm (M ± SD)	
Right	2.61 ± 0.90
Left	3.84 ± 0.93
Semen, median (IQR)	
Sperm count, million	22 (9–40)
Total motility, %	39 (14.5–56)
Progressive motility, %	10 (2–28)
Normal morphology, %	3 (1–6)
TMSC, %	18.96 (3.3–58.6)
sORP*, millivolt/10^6^ sperm/ml	3.03 (1.7–7.9)
Sperm DNA fragmentation (%)	25.0 (18.8–41)
Hormones (M±SD)	
Estradiol, pg/ml	103.40 ± 56.70
FSH, IU/L	4.60 ± 4.50
LH, IU/L	4.10 ± 2.90
Testosterone, nmol/L	17.70 ± 7.00

M ± SD: mean ± standard deviation; ^*a*^Measured by ultrasound; Max, maximum; IQR, inter quartile range; BMI, body mass index; TMSC, total motile sperm count; FSH, follicle stimulating hormone; LH, luteinizing hormone; sORP, static oxidation-reduction potential; *this analysis included 58 patients as it is not routinely undertaken for all patients.

### Pre-operative correlation between BMI and semen/hormone parameters

Table 2 shows that across the whole sample, BMI was significantly positively correlated with age but negatively correlated with sperm concentration, total motility, progressive motility and total motile sperm count. However, the strengths of the correlations were weak for all four parameters.

**Table 2.** Correlation between BMI and 12 study parameters (*N* = 813).

Parameter	Age	Semen							Hormones			
		Volume	Concentration	TM	PM	N morphology	TMSC	SDF	Estradiol	LH	FSH	TT
BMI	0.187	0.07	−0.196	−0.117	−0.107	−0.033	−0.143	−0.094	0.086	−0.007	0.056	−0.337
p-value	***<0.001***	***0.045***	***<0.0001***	***0.001***	***0.002***	0.262	***<0.0001***	0.371	***0.042***	0.593	0.592	***<0.0001***

LH, luteinizing hormone; TM, total motility; PM, progressive motility; N morphology, normal morphology; TMSC, total motile sperm count; SDF, sperm DNA fragmentation; italicized cells indicate statistical significance.

In terms of hormones, testosterone, estradiol, FSH, and LH levels were all within the normal range. BMI was significantly negatively correlated with serum testosterone level with a moderate strength of correlation and significantly positively correlated with serum estradiol level with a weak strength of correlation.

### Pre-operative semen and hormone levels across the BMI groups

Across the BMI groups, pre-operatively, there were significant differences in sperm concentration, total motility, progressive motility and total motile sperm count by BMI, where higher BMI groups (groups C and D) exhibited the poorest semen parameters (Table 3, pre-last column). Conversely, there were generally no differences in terms of pre-operative hormonal profiles by BMI, except for testosterone, where again, the highest BMI group (group D) exhibited the poorest testosterone levels (Table 3, pre-last column).

**Table 3.** Pre- and postoperative semen parameters, sperm DNA fragmentation values, and hormonal profile results by BMI.

Parameter	BMI (kg/m^2^)													
	Group A: <25 (*n* = 251)			Group B: 25–29 (*n* = 289)			Group C: 30–35 (*n* = 183)			Group D: >35 (*n* = 90)				
	Preop	Postop	P^∋^	Preop	Postop	P^∋^	Preop	Post-op	P^∋^	Pre-op	Post-op	P^∋^		
	Median (IQR)	Median (IQR)		Median (IQR)	Median (IQR)		Median (IQR)	Median (IQR)		Median (IQR)	Median (IQR)		P *	P **
**Semen**														
Volume (ml)	3 (2–4)	3 (2–4)	0.731	3 (2–4.5)	3 (2–4.5)	0.621	3 (2–4)	3 (2–4)	0.441	3 (2–4)	3 (2–4)	0.092	0.313	0.381
Concentration (million/ml)	28 (13.8–50)	30 (12.4–54)	***0.032***	20 (9–40)	26.8 (10–47.5)	***0.012***	19 (7–40)	25 (11.8–45.5)	***<0.001***	14.7 (4.8–26)	20.3 (4.1–36)	**0.011**	***<0.001***	0.912
Total Motility (%)	45 (21–60)	45.5 (30–62.7)	***0.006***	35 (12–54)	44 (14.3–60)	***<0.001***	35 (15–60)	44 (23–60)	***<0.001***	33 (11.5–50)	30 (13.8–61)	0.082	***0.003***	0.763
Progressive Motility (%)	13 (4–31)	23 (6–38)	***<0.001***	10 (2–27)	15 (3–33)	***0.041***	10 (1–24)	13 (5–35)	***<0.001***	6.5 (1–23.2)	10 (1–28)	0.332	***0.041***	0.342
Normal morphology (%)	4 (2–6)	4 (2–6)	0.092	3 (1–5)	4 (2–6)	***0.033***	4 (1–6)	3 (3–6)	0.378	3 (1–5)	3 (1–5)	0.291	0.123	***0.021***
Total Motile Sperm Count	29.8 (6.8–85.3)	37.1 (7.7–83.9)	0.084	17.8 (2.8–52.3)	28.5 (2.7–84.6)	***0.001***	15.4 (2.0–57.9)	32.6 (6.6–55.5)	***0.008***	15.5 (1.7–36.2)	19.7 (1.5–45.6)	0.813	***<0.001***	0.812
Sperm DNA Fragmentation (%)	27 (18–43)	29.5 (13.8–41.2)	0.224	26 (19.5–42)	38 (18–47.5)	0.652	22 (18.5–40)	29 (18.5–42.4)	0.971	22 (17–35)	36 (26–41)	0.674	0.413	0.632
**Hormones**														
Estradiol (pg/ml)	89 (69.2–114)	89 (63.7–115)	0.072	98 (72.5–126.7)	98 (76.5–125.5)	0.451	99 (73.7–124)	98 (71–122)	0.827	96.5 (66.5–134.3)	108 (84–133)	0.424	0.212	0.331
LH (IU/L)	4 (2.9–5)	3 (2.5–5)	0.232	4 (2.4–5)	3 (2–4)	0.456	3 (2–5)	3 (2–5)	0.232	4 (2.8–5)	3 (2–5)	0.263	0.532	0.571
FSH (IU/L)	3 (2–5)	3 (2–6)	0.764	3 (2–5.7)	3 (2–5)	0.691	4 (2–6.8)	4 (2–7.5)	0.414	4 (2–6)	4 (2–6)	0.952	0.563	0.921
Testosterone (nmol/L)	20.6 (15.7–25.1)	19.2 (14.8–25)	0.972	16 (12.8–20)	15.6 (13–20)	0.526	15.8 (12.3–20)	14.4 (12–18.8)	0.814	14.3 (11.2–15.8	12.9 (9.2–17.1)	0.491	***<0.001***	0.301

IQR: interquartile range; Pre-op: preoperative, Post-op: postoperative: FSH: follicle stimulating hormone; bold italicized cells indicate statistical significance; LH: luteinizing hormone.

^∋^Wilcoxon signed ranks test.

*Comparison of preoperative values: *p* values of the differences between preoperative values across all BMI groups (Kruskal-Wallis test).

**Comparison of extent of change (∆) before and after varicocelectomy: *p* values for extent of change for the given parameter across BMI groups (Kruskal-Wallis test).

### Post-operative semen and hormone levels of individual BMI groups

Table 3 also shows that post-operatively, in terms of semen quality, each of the four BMI groups showed significant improvement in sperm concentration compared to its pre-operative value. However, total and progressive motility were significantly improved in Groups A, B and C, whereas for Group D (highest BMI), total motility was improved clinically but did reach statistical significance, and progressive motility did not show improvement. Morphology was significantly improved only in Group B. Total motile sperm count was significantly improved only in Groups B and C. As regards the hormones, none of the four BMI groups showed significant changes in hormonal profile compared to its pre-operative value.

### Post-operative semen and hormone levels across the BMI groups

Table 3 further depicts the significance of the changes in semen and hormones after varicocelectomy across the four BMI groups. For semen parameters, the extent of change was not significantly different across the four BMI groups except for normal morphology, where postoperative improvement in morphology was most pronounced among Group B (25–29 BMI group) (Table 3, last column). With regards to the hormonal profile, the extent of post-operative change was not significantly different across the four BMI groups i.e. BMI did not influence the changes. sORP results were recorded for only 58 patients preoperatively and 23 patients postoperatively, thus statistical analysis couldn’t be performed between pre- and postoperative sORP data.

### Independent predictors of pre- and post-operative semen quality

Table 4 depicts the linear regression analysis of age, BMI and maximum vein diameter on pre- and post-operative semen quality reflected by total motile sperm count. Regression of pre-operative semen quality showed that age and maximum vein diameter were not independently associated with total motile sperm count while higher BMI was a negative independent predictor of total motile sperm count. Regression of post-operative data showed that higher BMI and larger maximum vein diameter positively and negatively predicted total motile sperm count respectively, while age was not associated with post-operative total motile sperm count.

**Table 4.** Linear regression analysis of age, BMI and maximum vein diameter on pre- and post-operative semen quality.

	Total motile sperm count					
	Pre-operative			Post-operative		
Variable	β	95% CI	p-value	β	95% CI	p-value
Age	0.025	(−0.94; 0.98)	0.956	−1.05	(−2.2; 0.09)	0.072
BMI	−2.56	(−3.92; −1.19)	***<0.0001***	−2.19	(−3.9; 0.47)	***0.013***
Maximum vein diameter	7.45	(−0.85; 15.75)	0.079	14.36	(4.02; 24.69)	***0.007***

BMI, body mass index; CI, confidence interval; bold italicized cells indicate statistical significance.

Table 5 summarizes the pre- and post-operative changes in semen parameters, and hormonal profile by and also across BMI groups.

**Table 5.** Summary of pre- and post-operative changes in semen parameters, and hormonal profile by and across BMI groups.

Parameter	Pre-varicocelectomy	Post-varicocelectomy
**Semen**		
Each individual BMI group	—	Improvement in most parameters, except for TM and PM
Across BMI groups	Worse with increase in BMI*	No significant differences in the changes, except for % NM**
**Hormone**		
Each individual BMI group	—	No changes
Across BMI groups	Lowest TT level in the highest BMI group*	No differences**

— not applicable; TT, testosterone; TM, total motility; PM, progressive motility; NM, normal morphology; *based on comparison of pre-operative variables across BMI groups; **based on extent of change (∆) in variables before and after varicocelectomy across BMI groups.

## Discussion

The subfertility among males resulting from varicocele and obesity is due to similar pathophysiological mechanisms [2,6]. Despite this, very few studies have assessed the possible association between obesity (BMI) and reproductive potential after varicocelectomy [12,14–17], and the findings of these very few studies were varied and inconclusive. This inconsistency incited us to examine the association of BMI with semen parameters and reproductive hormones before and after microsurgical varicocelectomy among a large sample of infertile men (*N* = 813).

Our main findings were that, preoperatively, obesity was associated with lower grades varicocele, lower levels of sperm concentration, total motility, progressive motility and serum testosterone level, and a higher level of serum estradiol level. Postoperatively, varicocelectomy resulted in: (a) improvement in most semen parameters for each BMI group, with no statistically significant difference across the different groups; and, (b) no changes in hormonal profile for each BMI group or across BMI groups. Higher BMI was a negative independent predictor of pre- and post-operative total motile sperm counts; age was not independent predictor; and larger maximum vein diameter was a positive independent predictor posto-perative total motile sperm count only.

Preoperatively, we found that the semen parameters including sperm concentration, total and progressive motility were significantly negatively correlated with the extent of obesity (i.e. BMI category), which is concordant with the literature [3–5]. Our observation that the post-varicocelectomy improvement in most semen parameters for each individual BMI group, with no statistically significant difference across the different BMI groups suggested that extent of obesity (BMI) did not play a role in the improvement of semen parameters after varicocelectomy. Our findings are in complete agreement with 3 previous studies, where the degree of post-operative improvements in semen parameters was not influenced by the extent of pre-operative obesity [13–15].

Conversely, another three studies found that preoperative BMI was significantly negatively correlated with postoperative semen parameters improvement, where individuals with higher preoperative BMI were less likely to exhibit improvement in semen parameters after varicocelectomy [11,12,17]. A point to note is that the samples of these three studies all comprised younger men (mean age 27.5, 26 and 22.5 years) than our sample’s mean age of 36 years. For instance, Abou Ghayada et al. showed that BMI was significantly negatively associated with postoperative semen parameters improvement only among younger men (<30 years) whereas older patients had no significant changes in postoperative semen parameters [16]. Collectively, such findings might propose that the inverse relationship between BMI and post-operative semen quality might be age-dependent, being more apparent at younger age groups. Such findings concur with two previous studies that showed better improvement in semen parameters among younger patients after varicocelectomy [26,27].

Other potential explanations why these studies [11,17] found a negative correlation between BMI and postoperative semen parameters improvement when the current study did not, could possibly be the surgery technique: use of loupe-assisted vs micro-surgical varicocelectomy technique. Evidence [28] have shown a higher chance of missing some veins in the former technique when compared with the latter. Such ‘missing’ could ‘dilute’ the positive therapeutic effect of varicocelectomy on semen. In addition, it is also plausible that missing the veins is also more likely to occur in technically challenging obese patients. Hence collectively the two factors of loupe-assisted technique and higher obesity could make an improvement in postoperative semen parameters less likely to be detected among the more obese patients, thus explaining the findings reported by other studies [11,17].

A point to note is that, in the present study, higher-grade varicocele was significantly more prevalent in lower BMI groups, and it is conceivable that such higher grades could have potentially incurred more insult to the semen production of the testis [29]. However, in the current study, the pre-operative semen parameters were significantly lower in the more obese patients (Groups C and D). This might be explained by the fact that in men with high BMI, other obesity-related pathophysiological mechanisms (e.g. increased OS, hyperthermia, hormonal imbalance) may amplify the effects of the varicocele leading to more deterioration of semen parameters. Another point to consider is that the evidence suggests that more pronounced recovery after varicocelectomy is seen with higher-grade varicocele [29]; and in addition, we did not find significant differences in the postoperative changes across the BMI groups, suggesting that grade of varicocele is not a confounding factor which was also confirmed in previous studies [14].

Regarding the hormonal profiles, our results showed that preoperatively, BMI was significantly negatively correlated with serum testosterone, and positively correlated with serum estradiol. However, postoperatively, there were no statistically significant changes in the hormones within each individual BMI group, and also between the four BMI groups. This suggests that lower testosterone and/or higher estradiol did not hinder post-operative improvements in semen parameters. Only two previous studies appraised the reproductive hormonal parameters before and after varicocelectomy [13,17]. The first study agrees with our findings and reported that preoperatively, there were significantly lower level of testosterone and higher level of estradiol among the obese group compared with the other groups, and the differences persisted postoperatively [17]. However, the second study showed that there was statistically significant post-operative increase in the serum testosterone in the whole sample [13] which is in concordance with some of the previously published research [30].

The current study has some limitations. The present study was in a single center inquiry, and retrospective studies inevitably suffer from the quality of data. In line with others, we followed up for 3 months [31], and a longer follow up would have been beneficial as improvement is semen parameters after varicocelectomy might require up to 6 months [32]. The reporting of the pregnancy rates would have been useful to confirm the actual fertility potential improvement. Further larger and prospective studies could address these limitations to unravel any effects of BMI as a factor on the improvement in semen parameters and fertility potential of sub fertile patients with clinical varicocele. The current study has many strengths. It was conducted in the only dedicated tertiary male infertility center in the country, and as we receive all the infertility cases, thus the current study is representative of the population of Qatar. The study also involved a sample size of patients many times larger than those of previous studies [11–17]. In order to reduce variability due to different laboratories [33], all study procedures (conventional and advanced semen tests, serum hormonal profile) were conducted in the same laboratory utilizing international standards of quality control, thus reflecting on the quality of reading of the semen parameters. Varicocelectomy was undertaken using microsurgical technique, considered the gold standard [34], and all surgical procedures were performed by same experienced urology team well versed in this procedure.

## Conclusion

Micro-surgical varicocelectomy is effective for infertile obese patients with clinical varicocele. It results in improvements in most of their semen parameters and might improve the fertility potential of such patients. BMI appears to have no influence on such effectiveness. Further larger and prospective studies are needed to rule out any possible impact of BMI on post varicocelectomy fertility potential.

## References

[1] Clarke BG. Incidence of varicocele in normal men and among men of different ages. JAMA. 1966;198(10):1121–1122. 5953394

[2] Arya D, Balasinor N, Singh D. Varicocele-associated male infertility: cellular and molecular perspectives of pathophysiology. Andrology. 2022;10(8):1463–1483. 36040837 10.1111/andr.13278

[3] Guo D, Wu W, Tang Q, et al. The impact of BMI on sperm parameters and the metabolite changes of seminal plasma concomitantly. Oncotarget. 2017;8(30):48619–48634. DOI:10.18632/oncotarget.14950 28159940 PMC5564712

[4] Hammoud A, Wilde N, Gibson M, et al. Male obesity and alteration in sperm parameters. Fertil Steril. 2008;90(6):2222–2225. DOI:10.1016/j.fertnstert.2007.10.011 18178190

[5] HI K, JB M, CW E, et al. Impact of body mass index values on sperm quantity and quality. J Androl. 2006;27(3):450–452. 16339454 10.2164/jandrol.05124

[6] Leisegang K, Sengupta P, Agarwal A, et al. Obesity and male infertility: mechanisms and management. Andrologia. 2021;53(1):e13617. DOI:10.1111/and.13617 32399992

[7] Jensen CFS, Østergren P, Dupree JM, et al. Varicocele and male infertility. Nat Rev Urol. 2017;14(9):523–533. DOI:10.1038/nrurol.2017.98 28675168

[8] Agarwal A, Cannarella R, Saleh R, et al. Impact of varicocele repair on semen parameters in infertile men: a systematic review and meta-analysis. World J Mens Health. 2022. DOI:10.5534/wjmh.220142 PMC10042659 36326166

[9] Majzoub A, ElBardisi H, Covarrubias S, et al. Effect of microsurgical varicocelectomy on fertility outcome and treatment plans of patients with severe oligozoospermia: an original report and meta-analysis. Andrologia. 2021;53(6):e14059. DOI:10.1111/and.14059 33763931

[10] Birowo P, Tendi W, Widyahening IS, et al. The benefits of varicocele repair for achieving pregnancy in male infertility: a systematic review and meta-analysis. Heliyon. 2020;6(11):e05439. DOI:10.1016/j.heliyon.2020.e05439 33204888 PMC7648199

[11] Kandevani NY, Namdari F, Hamidi M, et al. Developing a novel prediction model for the impact of varicocelectomy on postoperative fertility. Eur J Trans Myol. 2022;32(2):10411. DOI:10.4081/ejtm.2022.10411 PMC9295180 35502854

[12] Ates E, Ucar U, Keskin MZ, et al. Preoperative neutrophil-to-lymphocyte ratio as a new prognostic predictor after microsurgical subinguinal varicocelectomy. Andrologia. 2019;51(2):e13188. DOI:10.1111/and.13188 30397905

[13] Shabana W, Teleb M, Dawod T, et al. Predictors of improvement in semen parameters after varicocelectomy for male subfertility: a prospective study. Can Urol Assoc J. 2015;9(9–10):E579–582. DOI:10.5489/cuaj.2808 26425217 PMC4581921

[14] Chen S-S, Chen L. Predictive factors of successful varicocelectomy in infertile patients. Urol Int. 2011;86(3):320–324. 21212649 10.1159/000322825

[15] Pham KN, Sandlow J. The effect of body mass index on the outcomes of varicocelectomy. J Urol. 2012;187(1):219–221. 22100008 10.1016/j.juro.2011.09.033

[16] Abou Ghayda R, El-Doueihi RZ, Lee JY, et al. Anthropometric variables as predictors of semen parameters and fertility outcomes after varicocelectomy. J Clin Med. 2020;9(4):1160. DOI:10.3390/jcm9041160 32325696 PMC7230912

[17] El-Dighidy MA, Sherief MH, Shamaa MA, et al. Smoking and obesity negatively affect the favorable outcome of varicocelectomy in sub-fertile men. Andrologia. 2021;53(8):e14131. DOI:10.1111/and.14131 34117798

[18] Ahmad R, Haque M. Obesity: a doorway to a molecular path leading to infertility. Cureus. 2022;14(10):e30770. DOI:10.7759/cureus.30770. 36320802 PMC9612950

[19] Chaudhuri GR, Das A, Kesh SB, et al. Obesity and male infertility: multifaceted reproductive disruption. Middle East Fertil Soc J. 2022;27(1):8. DOI:10.1186/s43043-022-00099-2

[20] WHO. Physical status: the use and interpretation of anthropometry: report of a World Health Organization (WHO) expert committee. Geneva, Switzerland: World Health Organization; 1995. 8594834

[21] Cooper TG, Noonan E, von Eckardstein S, et al. World Health Organization reference values for human semen characteristics*‡. Human Reproduction Update. 2010;16(3):231–245. DOI:10.1093/humupd/dmp048 19934213

[22] Fernandez JL, Muriel L, Rivero MT, et al. The sperm chromatin dispersion test: a simple method for the determination of sperm DNA fragmentation. J Androl. 2003;24(1):59–66. 12514084

[23] Agarwal A, Roychoudhury S, Sharma R, et al. Diagnostic application of oxidation-reduction potential assay for measurement of oxidative stress: clinical utility in male factor infertility. Reprod Biomed Online. 2017;34(1):48–57. DOI:10.1016/j.rbmo.2016.10.008 27839743

[24] Agarwal A, Arafa M, Chandrakumar R, et al. A multicenter study to evaluate oxidative stress by oxidation-reduction potential, a reliable and reproducible method. Andrology. 2017;5(5):939–945. DOI:10.1111/andr.12395 28726302

[25] Majzoub A, Elbardisi H, Arafa M, et al. Does the number of veins ligated during varicocele surgery influence post-operative semen and hormone results? Andrology. 2016;4(5):939–943. DOI:10.1111/andr.12226 27317389

[26] Hassanzadeh-Nokashty K, Yavarikia,P, C, Hazhir, S, Hassanzadeh, M. Effect of age on semen parameters in infertile men after varicocelectomy. Ther Clin Risk Manag. 2011;7:333–6. DOI:10.2147/TCRM.S17027. Epub 2011 Aug 9. PMID: 21941438; PMCID: PMC3176165 21941438 PMC3176165

[27] Bolat MS, Kocamanoglu F, Gulsen M, Sengul, M, Asci, R. The impact of age on fertility rate in patients who underwent microsurgical varicocelectomy. Andrologia. 2019;51(4):e13234. DOI:10.1111/and.13234. Epub 2019 Jan 28. PMID: 30689241 30689241

[28] Zhang H, Liu X-P, Yang X-J, et al. Loupe-assisted versus microscopic varicocelectomy: is there an intraoperative anatomic difference? Asian J Androl. 2014;16(1):112–114. DOI:10.4103/1008-682X.122189 24369142 PMC3901867

[29] Asafu-Adjei D, Judge C, Deibert CM, et al. Systematic review of the impact of varicocele grade on response to surgical management. J Urol. 2020;203(1):48–56. DOI:10.1097/JU.0000000000000311 31042452

[30] Zohdy W, Ghazi S, Arafa M. Impact of varicocelectomy on gonadal and erectile functions in men with hypogonadism and infertility. J Sex Med. 2011;8(3):885–893. 20722780 10.1111/j.1743-6109.2010.01974.x

[31] Ghaed MA, Makian SA, Moradi A, Maghsoudi, R, Gandomi-, Mohammadabadi A, et al. Best time to wait for the improvement of the sperm parameter after varicocelectomy: 3 or 6 months? Arch Ital Urol Androl. 2020;92(3). DOI:10.4081/aiua.2020.3.259. PMID: 33016058 33016058

[32] Machen GL, Johnson D, Nissen MA, et al. Time to improvement of semen parameters after microscopic varicocelectomy: when it occurs and its effects on fertility. Andrologia. 2020;52(2):e13500. DOI:10.1111/and.13500 31840291

[33] Jorgensen N, Auger J, Giwercman A, et al. Semen analysis performed by different laboratory teams: an intervariation study. Int J Androl. 1997;20(4):201–208. DOI:10.1046/j.1365-2605.1997.00052.x 9401822

[34] Mehta A, Goldstein M. Microsurgical varicocelectomy: a review. Asian J Androl. 2013;15(1):56–60. 23147467 10.1038/aja.2012.98 PMC3739135

